# Planktonic, benthic and sympagic copepods collected from the desalination unit of Mario Zucchelli Research Station in Terra Nova Bay (Ross Sea, Antarctica).

**DOI:** 10.3897/BDJ.12.e119633

**Published:** 2024-03-11

**Authors:** Marco Grillo, Guido Bonello, Matteo Cecchetto, Alice Guzzi, Nicholas Noli, Valentina Cometti, Stefano Schiaparelli

**Affiliations:** 1 Italian National Antarctic Museum (MNA, section of Genoa), Genoa, Italy Italian National Antarctic Museum (MNA, section of Genoa) Genoa Italy; 2 Department of Physical Sciences, Earth and Environment (DSFTA) - University of Siena, Siena, Italy Department of Physical Sciences, Earth and Environment (DSFTA) - University of Siena Siena Italy; 3 GeoScape Society Cooperative, Genoa, Italy GeoScape Society Cooperative Genoa Italy; 4 Department of Earth, Environmental and Life Sciences (DISTAV) - University of Genoa, Genoa, Italy Department of Earth, Environmental and Life Sciences (DISTAV) - University of Genoa Genoa Italy

**Keywords:** Terra Nova Bay, Ross Sea, Museum collection, coastal ecosystem

## Abstract

**Background:**

Distributional data on planktonic, benthic and sympagic copepods collected in the framework of the XXXIV^th^ Expeditions of the Italian National Antarctic Programme (PNRA) to the Ross Sea sector from 2018–2019 are here provided. These occurrences correspond to specimens collected from the 25 μm filters used in the desalination plant of the Italian research station "Mario Zucchelli" (MZS), located in the Terra Nova Bay area (TNB; Ross Sea, Antarctica). This dataset is a contribution to the Antarctic Biodiversity Portal, the thematic Antarctic node for both the Ocean Biogeographic Information System (AntOBIS) and the Global Biodiversity Information Facility Antarctic Biodiversity Information Facility (ANTABIF). The dataset was uploaded and integrated with the SCAR-AntOBIS database (the geospatial component of SCAR-MarBIN). Please follow the guidelines from the SCAR Data Policy (ISSN 1998-0337) when using the data. If you have any questions regarding this dataset, please contact us via the contact information provided in the metadata or via data-biodiversity-aq@naturalsciences.be. Issues with the dataset can be reported at the biodiversity-aq GitHub project.

**New information:**

We describe the diversity of marine copepods Terra Nova Bay sampled by the filters installed in the desalination unit (DU) of the Italian research station "Mario Zucchelli" described in recent work. The opening of the intake pipe of the DU is positioned at a depth of 4 m and allowed a total of 2,116 specimens to be sampled and recognised. In addition, new occurrence records of copepod genera and species are reported in the same zone. We provide an overview of the marine copepod diversity reported for TNB. The total of 2,116 individuals corresponds to 14 genera and 15 species and is represented by 136 occurrence records in this dataset. Around 52% of the total number of species are new records for the TNB area. The publication of this data paper was funded by the Belgian Science Policy Office (BELSPO, contract n°FR/36/AN1/AntaBIS) in the Framework of EU-Lifewatch as a contribution to the SCAR Antarctic biodiversity portal (biodiversity.aq).

## Introduction

Copepoda are a major component of zooplankton assemblages and are a fundamental class in marine food webs, representing 70% of the mesozooplankton biomass (Carli et al. 2000). These organisms can be found in different ecological categories, such as neuston (Zaitsev 1971, Maki and Herwig 1991), plankton (Kim et al. 2022) and benthos (Stark et al. 2020) and have different trophic strategies (e.g. predators, filter feeders, parasites, suspension feeders) (Boxshall and Halsey 2004, Michels and Schnack-Schiel 2005). There are currently 302 planktonic copepod species in Antarctica (Razouls et al. 2022) whose distribution was recently reassessed (De Broyer et al. 2014).

Copepod communities are important in trophodynamic terms for secondary production and the grazing effect (Atkinson 1996, Hansen et al. 1997, Calbet and Landry 2004). These crustaceans represent a fundamental food web link between marine primary producers and higher consumers (Pakhomov et al. 2020), such as cnidarians, fish, seabirds and even mammals (Atkinson 1998, Turner 2004).

Their reaction to changes in environmental conditions (e.g. modifications in water column stratification and water acidification (Barton et al. 2013)) triggered by climate change is known, which may result in changes in their distribution, life cycle (Poloczanska et al. 2013) and physiological adaptations as reported by recent scientific investigations (Kim et al. 2022). Copepod assemblages represent a good environmental indicator (Edwards and Richardson 2004, Hays et al. 2005, Edwards 2009) to pinpoint and evaluate environmental changes and global and anthropogenic-made climate changes (Turner 2004, Bonello et al. 2018, Bonello et al. 2022).

Copepod communities in the Ross Sea area have been extensively studied since 1985 and were part of the objectives of the first Italian Ocenographic Expeditions of the PNRA (Amato 1990). The scientific team of those expeditions studied the biodiversity and ecological roles of planktonic copepods (Carli et al. 1989, Carli et al. 1990, Zunini Sertorio et al. 1990, Guglielmo et al. 1990, Zunini Sertorio et al. 1992, Bonello et al. 2020, Carli et al. 2000, Zunini Sertorio et al. 2000, Carli et al. 2002, Pane et al. 2004, Grillo et al. 2022) and their association with pack-ice (Guglielmo et al. 2007, Granata et al. 2009, Guglielmo et al. 2015, Granata et al. 2022); however, to date, information regarding the diversity of benthic copepods is still scarce.

In Bonello et al. (2020), a total of 8,224 specimens of Antarctic copepods are reported, after the analysis of materials from the III^rd^, V^th^ and X^th^ Italian Antarctic expeditions, which led to the production of the first checklist for this taxon in the area. This checklist, in addition to the physical samples currently deposited in the biological collection of the Italian National Antarctic Museum (MNA), contains the digitised data, mostly belonging to grey literature, recovered from the PNRA expedition reports. The authors digitised campaigns and distribution data for each copepod species, resulting in a copepod community historical baseline for future research comparison. During the XXXIV^th^ PNRA expedition (2018–2019), neritic copepod diversity obtained from the DU filters of the Italian research base “*Mario Zucchelli*” (MZS) (Terra Nova Bay, Ross Sea) was collected from the desalination plant. The use of DU as a sampling method has already been applied for the study of nanoplankton (Cecchetto et al. 2021), picoplankton, phytoplankton (Balzano et al. 2015) and invertebrate larval stages (Heimeier et al. 2010a, Heimeier et al. 2010b). Here, we report the copepod samples collected using this sampling technique during that expedition, from 29 December 2018 to 02 February 2019.

Previous MNA contributions focused on Mollusca, Tanaidacea, Fungi, Ophiuroidea, Porifera, Bryozoa, Rotifera, Asteroidea and Copepoda (Ghiglione et al. 2013, Piazza et al. 2014, Selbmann et al. 2015, Cecchetto et al. 2017, Ghiglione et al. 2018, Cecchetto et al. 2019, Bonello et al. 2020, Garlasché et al. 2020, Guzzi et al. 2022). The special issue that included this publication contains additional articles that centre on specific marine animals, such as Holothurians (Guzzi et al., in prep.), Amphipods (Cecchetto et al., in prep.), Isopods (Noli et al., in prep.), fouling ARMS (Cometti et al., in prep.) and fish. This dataset also represents another Italian contribution to the CCAMLR CONSERVATION MEASURE 91-05 (2016) for the Ross Sea region Marine Protected Area, specifically addressing Annex 91-05/C (“long-term monitoring of benthic ecosystem functions”).

## Project description

### Title

Planktonic, benthic and sympagic copepods collected in the desalination unit during the XXXIV^th^ Expedition of the Italian National Antarctic Program (PNRA).

### Personnel

Grillo Marco, Bonello Guido, Cecchetto Matteo, Guzzi Alice, Noli Nicholas, Cometti Valentina, Schiaparelli Stefano.

### Study area description

**Figure 1.**
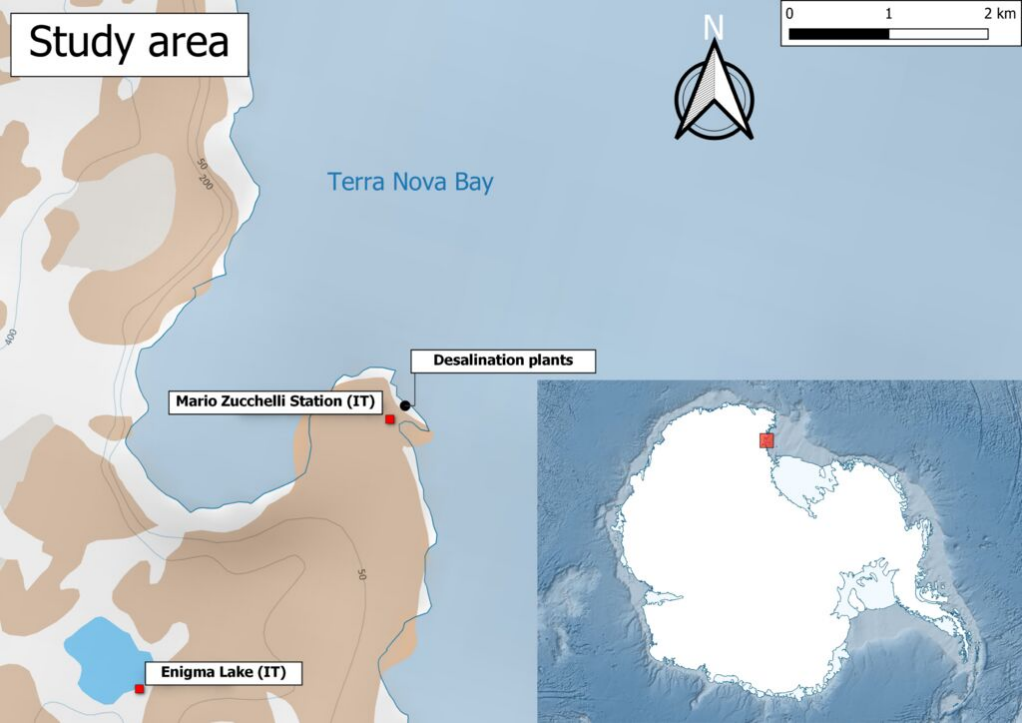
Location of the desalination plant intake pipe (black circle).

The distributional data of the copepods studied in this data paper derives from the XXXIV^th^ PNRA Antarctic expedition (Fig. 1). The seawater intake pipeline of the desalination plant (−74.693°, 164.118°) opens at a depth of 4 m in the locality of "Punta Stocchino." "Punta Stocchino" is located on the rocky promontory facing MZS and is about 200 m long. This area is located in the centre of Terra Nova Bay, which is located between the Drygalski Ice Tongue and the Cape Washington Penisula. The sampling timeframe was between 29 December 2018 and 2 February 2019.

### Funding

Data originated in the framework of the PNRA XXXIV^th^ Antarctic Expeditions (2018–2019) within the PNRA-funded research projects ”TNB-CODE" - Barcoding e metabarcoding di organismi Antartici marini, terrestri e limnetici”. Mario Zucchelli Station (Project code PNRA 2016/AZ1.17; PI Prof. Schiaparelli S.) and "RosS-MODe“ - Ross Sea biodiversity Monitoring through barcoding, metabarcODing and e-DNA” (Project code PNRA 18_00078; PI Prof. Ficetola F.).

The Italian National Antarctic Museum (MNA) hired two experts, G. Bonello and M. Grillo, with research contracts #2993 and #2992 issued on 25 June 2019, to analyse and identify to the lowest possible taxonomic resolution which the specimens represent in the samples.

The publication of this data paper was funded by the Belgian Science Policy Office (BELSPO, contract n°FR/36/AN1/AntaBIS) in the Framework of EU-Lifewatch as a contribution to the SCAR Antarctic biodiversity portal .

## Sampling methods

### Sampling description

**Figure 2.**
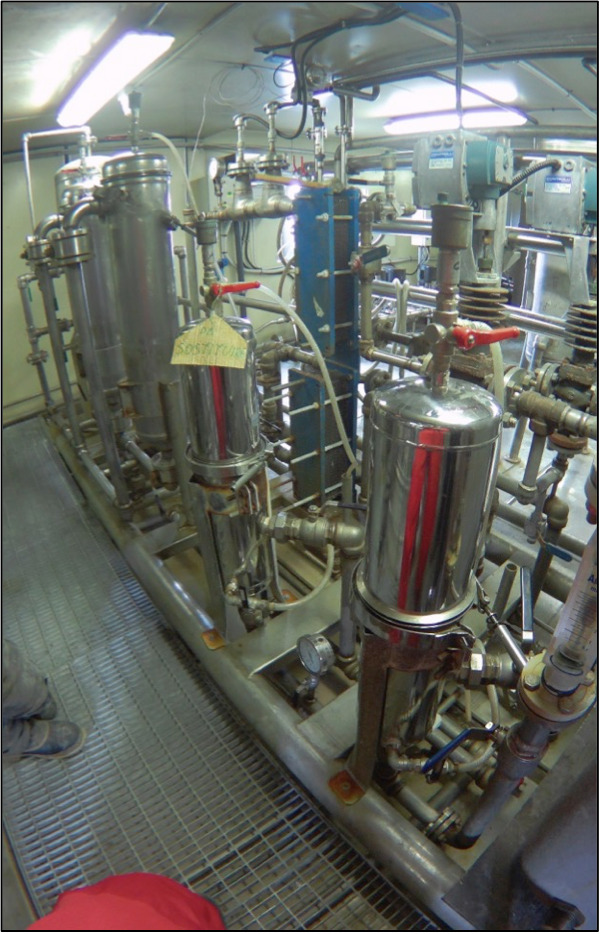
Desalination unit of Mario Zucchelli Station.

Samples were collected using the DU plant of MZS (Fig. 2), whose intake pipe is located at 4 m of depth in the locality of "Punta Stocchino". This plant is used to provide freshwater for the research base's activities, operating during the entire expedition’s summer season, generally from mid-October to the beginning of February. From the seawater intake pipe, a series of pipes and valves allow the water to flow to the main structure of the plant, located inside the research station, where the first steps of filtration (called “pre-filtration”) are conducted. These steps consist of a series of disposable filters positioned sequentially with a decreasing mesh size. The first one is packed with anthracite, followed by polyester bag filters of 25 μm mesh size and, finally, by polypropylene cartridges of 5 μm mesh size. The samples reported in this dataset were obtained from the biological material recovered by the 25 μm mesh size filters. More information on the technical specifications of the MZS DU plant can be found in Cecchetto et al. (2021).

### Quality control

All records were validated. Coordinates were converted into decimal latitude and decimal longitude and plotted to verify the geographical location and locality. All scientific names were checked for typos and matched to the species information backbone of Worlds Register of Marine Species and AphiaID was assigned to each taxon as scientificNameID. The event date and time were converted into ISO 8601 and verified with the field reports.

### Step description

**Figure 3.**
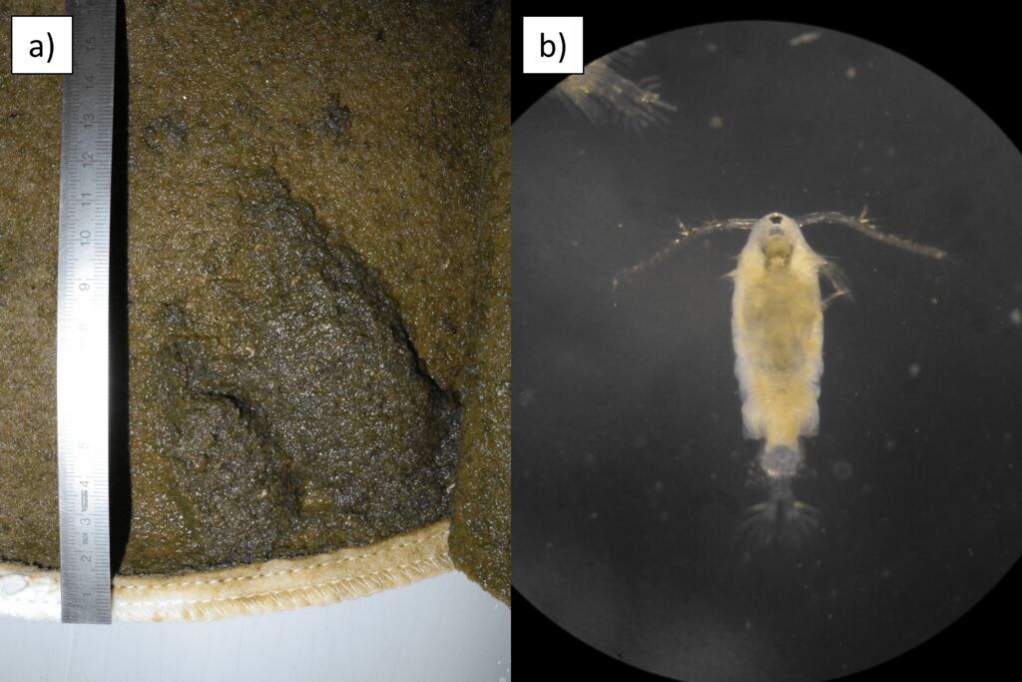
Filter bag (25 µm mesh) with bulk filtered biological material. a) Detail of the open lower portion of the filter bag; b) *Paralabidoceraantarctica* (Thompson I.C., 1898) found in the filter.

The 25 μm mesh size filters are replaced by the DU plant’s technician as soon as the pressure inside the filter housing reaches warning levels to prevent the clogging of the system. After removing the filters from their respective housings, the same were transported to the laboratory and processed following Cecchetto et al. (2022). Briefly, the filters, after removing the metal ring placed at the opening of the filter, were cut longitudinally in order to access their content, i.e. the biological material filtered (Fig. 3). Using a scalpel with sterilised, disposable blades, different cuts were performed in different positions of the filter and stored at −20°C, obtaining pieces of the filter that would later be used for metagenomic research purposes. From the remaining parts of the filter, depending on the amount of biological material present on the filter’s surface, different 15-ml Falcon tubes of material were scooped from the filter’s surface using a sterilised spatula and all the materials treated were then brought to volume with 96% ethanol. The Falcon tubes contained a mix of phytoplanktonic and zooplanktonic organisms in different ratios, depending on the biological community that was present in the water column facing the DU intake pipe during the filters’ operating time. The samples, stored at +4°C, were shipped to the MNA (Genoa section) laboratories, where the content of the Falcon tubes was sorted and analysed.

The collected copepods were counted and identified at the lowest possible level by GB and MG, based on morphological examination and by considering historic and recent bibliography (e.g. Bonello et al. (2020), Boxshall and Halsey (2004)). The online portals World Registry of Marine Species (WoRMS) and Banyuls sur Mer marine Copepoda database (Razouls et al. 2022) were used to confirm the acceptance of species names. When identification was inconclusive, only genus or family names were assigned. For the specimens recognised in this dataset, selected individuals were used to produce high-resolution images of morphological characters useful to species classification. Various acquisition techniques were performed to obtain these photos, such as scanning electron microscopy (SEM) and fluorescence microscopy with different colourations (Congo Red and Fuchsin) (Michels and Büntzow 2010, Ivanenko et al. 2012).

The original unsorted plankton matrix is stored in 96% ethanol and refrigerated at −20°C. The copepod specimens, split, sorted and identified, are in 96% ethanol or fixed on a slide and permanently deposited in the biological collection of the MNA with a specific MNA voucher number (from MNA-13263 to MNA-13174, from MNA-13276 to MNA-13278, from MNA-13743 to MNA-13748, MNA-13754, from MNA-13764 to MNA-13768, from MNA-15192 to 15197, from MNA-15199 to MNA-15250, MNA-15252, MNA-15253, MNA-15624 and MNA-15625). Antarctic copepod distribution data have been uploaded to the GBIF portal.

**Figure 4.**
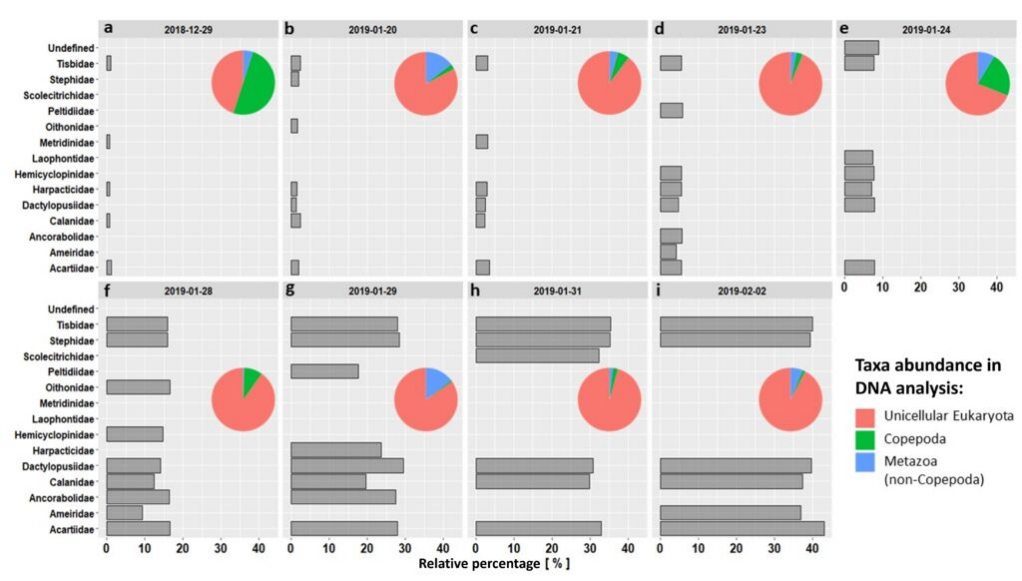
Percentage variation of occurrences by copepod family (bar graph) during the sampling period and the relative percentage composition of taxa obtained from DNA analysis (pie chart).

A metabarcoding methodology was also applied to the DU plant’s filters and only some preliminary and qualitative results are here reported. Specifically, the relative abundance of 18S rRNA sequences identified by the taxonomic identification of the metabarcoding protocol as copepods with respect to the total number of sequences is here reported only to illustrate the temporal dynamics that could be discerned by the metabarcoding approach during the sampling period (Fig. 4).

## Geographic coverage

### Description

Samples were collected at one location, the Italian “Mario Zucchelli” research station (MZS) in Terra Nova Bay (TNB) (Ross Sea, Antarctica) (Fig. 1), over several days.

Coordinates of desalination unit: −74.693° latitude; 164.118° longitude.

## Taxonomic coverage

### Description

The Copepoda diversity of the dataset is displayed in a total of 167 MNA vouchers (comprising vials with single species isolated from bulk samples and glass slides with dissected or whole specimens) collected during nine different sampling days (i.e. when filters have been changed). A total of 2,116 individuals were obtained, with Harpacticoida representing the most frequent order (52.1%), followed by Calanoida (44.3%) and Cyclopida (3.6%).

**Figure 5.**
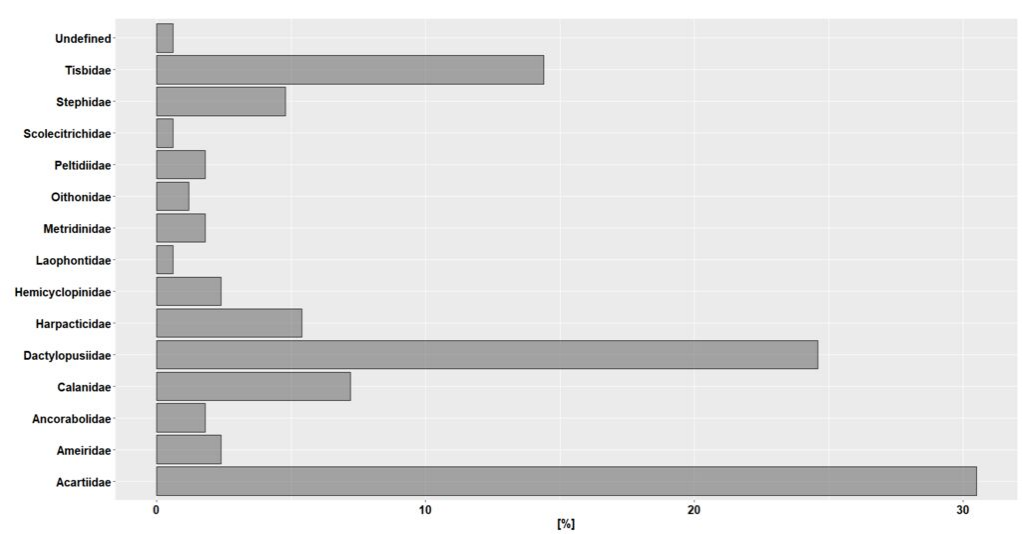
Diversity and relative frequency percentage at the family level for the number of individuals in the dataset.

Copepod species sampled via the DU consist of 14 families (Fig. 5), 17 genera and 14 species with 49 morphotypes that could not be identified and indicated as "sp." or "spp." in the dataset.

The most frequent families were Acartiidae (30.53%), Dactylopusiidae (24.55%) and Tisbidae (14.37%), while less frequent families have been Calanidae (7.18%), Harpacticidae (5.38%), Stephidae (4.79%), Ameiridae (2.40%), Hemicyclopinidae (2.40%), Ancorabolidae (1.80%), Metridinidae (1.80%), Peltidiidae (1.80%), Oithonidae (1.20%), Laophontidae (0.60%) and Scolecitrichidae (0.60%) and undefined (0.60%) (Fig. 5).

Regarding the life stages of the specimens, the dataset is composed of a majority of adults (94%), followed by the copepodite stages (6%).

From the literature review, the copepods found inside the DU samples can, generally, be assigned to the following habitats: benthos (47.90%), ice (35.33%), plankton (10.78%), benthos/ice (5.39%); the remaining 0.6% could not be assessed and are reported as unidentified. Fig. 4 shows, for each sampling date, the percentage variation of occurrences by copepod family (bar graph) and the percentage taxonomic composition obtained from DNA analysis (pie chart). Species and genera with the symbol (*) in the following table indicate that they represent new records for the TNB site.

### Taxa included

**Table:** 

Rank	Scientific Name	
kingdom	Animalia	
phylum	Arthropoda	
class	Maxillopoda	
order	Calanoida	
order	Cyclopoida	
order	Harpacticoida	
family	Acartiidae	
family	Ameiridae	
family	Ancorabolidae	
family	Calanidae	
family	Dactylopusiidae	
family	Harpacticidae	
family	Hemicyclopinidae	
family	Laophontidae	
family	Metridinidae	
family	Oithonidae	
family	Oncaeidae	
family	Peltidiidae	
family	Scolecitrichidae	
family	Stephidae	
family	Tisbidae	
genus	*Alteutha* Baird, 1846 *	
genus	*Ameira* Boeck, 1865*	
genus	*Calanoides* Brady, 1883	
genus	*Calanus* Leach, 1816	
genus	*Dactylopusia* Norman, 1903*	
genus	*Harpacticus* Milne Edwards H., 1840	
genus	*Laophonte* Philippi, 1840	
genus	*Laophontodes* Scott T., 1894*	
genus	*Lophotrix* Giesbrecht, 1895*	
genus	*Metridia* Boek, 1865	
genus	*Oithona* Braird, 1843	
genus	*Paradactylopodia* Lang, 1944	
genus	*Paralabidocera* Wolfenden, 1908	
genus	*Pseudocyclopina* Lang, 1946*	
genus	*Stephos* Scott T., 1892	
genus	*Tisbe* Lilljeborg, 1853	
species	*Alteuthadepressa* (Bairf, 1837)*	
species	*Calanoidesacutus* (Giesbrecht, 1902)	
species	*Calanuspropinquus* Brady, 1883	
species	*Dactypusiatisboides* (Claus, 1863)*	
species	*Harpacticusfurcifer* Giesbrecht, 1902	
species	*Laophonteglacialis* Brady, 1910	
species	*Laophontodestypicus* Scott T., 1894*	
species	*Metridiagerlachei* Giesbrecht, 1902	
species	*Oithonasimilis* Claus, 1866	
species	*Paradactylopodiabrevicornis* (Claus, 1866)*	
species	*Paralabidoceraantarctica* (Thompson I.C., 1898)	
species	*Pseudocyclopinaberndtreyi* Elwers, Martínez Arbizu & Fiers, 2001*	
species	*Stephoslongipes* Giesbrecht, 1902	
species	*Tisbegracilipes* Scott T., 1912	

## Temporal coverage

### Notes

29 December 2018 to 02 February 2019.

## Collection data

### Collection name

MNA - Biological Collections

### Collection identifier

https://www.gbif.org/grscicoll/collection/a57a1dc1-706c-42db-bbad-1e68d9685439

### Parent collection identifier

Italian National Antarctic Museum (section of Genoa)

### Specimen preservation method

specimens in jars in 96% ethanol, slides with whole or dissected organisms (fixed in glycerol) and frozen at −20°C.

## Usage licence

### Usage licence

Other

### IP rights notes

The dataset was published under the licence CC-BY 4.0.

## Data resources

### Data package title

Planktonic, benthic and sympagic copepods collected in the desalination unit during the XXXIV^th^ Expedition of the Italian National Antarctic Programme (PNRA)

### Resource link

https://doi.org/10.15468/uhzqru

### Alternative identifiers

https://ipt.biodiversity.aq/resource?r=mna_planktonic-benthic-sympagic-copepod

### Number of data sets

1

### Data set 1.

#### Data set name

Planktonic, benthic and sympagic copepods collected in the desalination unit during the XXXIV^th^ Expedition of the Italian National Antarctic Programme (PNRA).

#### Data format

Darwin Core

#### Description

This dataset is built on information from the copepod specimens analysed in this work. The aims and objectives of the XXXIVth PNRA can be found in the related campaign report (Melchiori 2019). The samples were pooled into a single dataset. This dataset will be useful to investigate the community structure of zooplankton and their relative larval stages.

**Data set 1.** 

Column label	Column description
occurrenceID	A global unique identifier for the Occurrence (as opposed to a particular digital record of the occurrence).
institutionCode	The name (or acronym) in use by the institution having custody of the object(s) or information referred to in the record.
instituitonID	An identifier for the institution having custody of the object(s) or information referred to in the record.
collectionCode	The name, acronym, coden or initialism identifying the collection or dataset from which the record was derived (as shown on the Global Registry of Scientific Collections).
collectionID	An identifier for the collection or dataset from which the record was derived.
catalogNumber	An identifier of any form assigned by the source within a physical collection or digital dataset for the record which may not be unique, but should be fairly unique in combination with the institution and collection code.
basisOfRecord	The specific nature of the data record and is here always reported as “PreservedSpecimen”.
type	Defines the nature of the resource, here is always “PhysicalObject”.
scientificName	The identification at the lowest taxonomic rank, without authorship information.
TaxonRank	The taxonomic rank of the most specific name in the scientificName.
kingdom	The full scientific name of the kingdom in which the taxon is classified.
phylum	The full scientific name of the phylum in which the taxon is classified.
class	The full scientific name of the class in which the taxon is classified.
order	The full scientific name of the order in which the taxon is classified.
family	The full scientific name of the family in which the taxon is classified.
genus	The full scientific name of the genus in which the taxon is classified.
specificEpithet	The name of the first or species epithet of the scientificName.
scientificNameAuthorship	The authorship information for the scientificName formatted according to the conventions of the applicable.
identificationQualifier	Abrief phrase or a standard term (sp., spp.) to express the determiner's doubts about the Identification.
scientificNameID	The globally unique identifier for the taxonomic information related to the scientificName and stored in WoRMS, the AphiaID.
individualCount	The number of individuals present.
sex	The sex of the identified specimens.
lifeStage	The life stage of organisms. In detail: CI: copepodite I, CII: copepodite II, CIII: copepodite III, CIV: copepodite IV; CV: copepodite V.
occurrenceRemarks	Campaign in which the organisms were sampled.
eventDate	Date the organisms were sampled.
year	Sampling year.
month	Sampling month.
day	Sampling day.
eventID	Unique code with data relating to the campaign and sampling date.
decimalLatitude	The geographic latitude (in decimal degrees, using the spatial reference system given in geodeticDatum).
decimalLongitude	The geographic longitude (in decimal degrees, using the spatial reference system given in geodeticDatum).
geodedicDatum	Spatial reference system (WGS84) upon which the geographic coordinates given in decimalLatitude and decimalLongitude are based.
minimumDepthInMetres	Minimum sampling depth during event in metres.
maximumDepthInMetres	Maximum sampling depth during event in metres.
samplingProtocol	Gear used to collect specimens and relative DOI of manuscript in which the sampling method is described.
eventRemarks	Filter number of the desalinisation unit plants.
preparations	Alist of preparations and preservation methods for a specimen. In detail: whole organism (96% ethanol), whole organism (slide fixed in glycerol) and dissected organism (slide fixed in glycerol).
taxonRemarks	Remarks on taxa, in this case, which ecological category the analysed species occupy.
coordinateUncertaintyInMetres	Horizontal distance, measured in metres, between the given decimal latitude and decimal longitude represents the radius of the minimum circle that includes the entire area.
occurrenceStatus	Astatement about the presence or absence of a specimen.
continet	Continent where the organisms were sampled.
countryCode	The standard code for the country where the organisms were sampled.
recordedBy	Surname and name of the personnel who collected the samples.
recordedByID	ORCID of the personnel who collected the samples.
identifiedBy	Surname and name of the personnel who analysed and recognised the single species.
identifiedByID	ORCID of the personnel who analysed and recognised the single species.
coordinatePrecision	A decimal representation of the precision of the coordinates given in the decimalLatitude and decimalLongitude.
